# Current Options in the Valorisation of Vine Pruning Residue for the Production of Biofuels, Biopolymers, Antioxidants, and Bio-Composites following the Concept of Biorefinery: A Review

**DOI:** 10.3390/polym14091640

**Published:** 2022-04-19

**Authors:** Meirielly Jesus, Aloia Romaní, Fernando Mata, Lucília Domingues

**Affiliations:** 1 CISAS—Center for Research and Development in Agri-Food Systems and Sustainability, Polytechnic Institute of Viana do Castelo, Rua da Escola Industrial e Comercial Nun’Alvares 34, 4900-347 Viana do Castelo, Portugal; fernandomata@ipvc.pt; 2 Chemical Engineering Department, Faculty of Science, University of Vigo, 32004 Ourense, Spain; aloia@uvigo.es; 3 CEB—Centre of Biological Engineering, University of Minho, Campus Gualtar, 4710-057 Braga, Portugal; 4 LABBELS—Associate Laboratory, 4710-057 Braga, Portugal

**Keywords:** vine pruning residue, biorefinery concept, pre-treatment, integral valorisation

## Abstract

Europe is considered the largest producer of wine worldwide, showing a high market potential. Several wastes are generated at the different stages of the wine production process, namely, vine pruning, stalks, and grape marc. Typically, these residues are not used and are commonly discarded. Portugal generates annually approximately 178 thousand metric tons of wine production waste. In this context, the interest in redirecting the use of these residues has increased due to overproduction, great availability, and low costs. The utilization of these lignocellulosic biomasses derived from the wine industry would economically benefit the producers, while mitigating impacts on the environment. These by-products can be submitted to pre-treatments (physical, chemical, and biological) for the separation of different compounds with high industrial interest, reducing the waste of agro-industrial activities and increasing industrial profitability. Particularly, vine-pruning residue, besides being a source of sugar, has high nutritional value and may serve as a source of phenolic compounds. These compounds can be obtained by bioconversion, following a concept of biorefinery. In this framework, the current routes of the valorisation of the pruning residues will be addressed and put into a circular economy context.

## 1. Introduction

Nowadays, environmental concerns and dependence on fossil resources encourage the scientific community to devote great efforts to the pursuit of renewable sources of energy for the manufacture of products. Strategies and policies for the development of sustainable growth based on the efficient use of natural resources are one of the main targets to achieve a circular bioeconomy in compliance with the sustainable development goals of Agenda 2030 [1]. In fact, an increase in biomass demand for bioenergy and biomaterials requirements is expected by 2050 as the result of the growing population [2]. One of the main sources of renewable biomass is lignocellulosic materials due to their high regeneration capacity and the fact that they are the most widespread and abundant carbon source in the world [3].

Lignocellulosic materials are composed of 0–10% non-structural (including ashes, extracts, other components in minor amounts) and structural compounds (40–50% of cellulose, 15–25% of hemicellulose, and 15–25% of lignin) [4,5]. The source of lignocellulosic materials can include the residues of forestry or crop production, as well as energy crops that use the whole plant biomass [6]. Residues from agro-food activities represent a large part of the lignocellulosic biomass generated around the world. The waste management associated with these activities is normally related to a negative environmental impact since some of these residues are burned, releasing unhealthy compounds [7]. In addition, these residues are enriched with bio-based compounds, with potential interest for the industry. Therefore, the valorisation of these residues could contribute to attaining a sustainable industrial sector through an alternative scheme of processing [8].

Among food industries, the winemaking sector represents one of the most important in the world. In this context, 7.5 million ha of vineyards were dedicated to grape crops, reaching a production of 73.3 million metric tons in 2017. The European Union has stood out in the world production of grapes (37%) and wine (279 million hL). Among the European countries, Italy (15.9%), France (16.4%), and Spain (12.1%), where the wine industry is an important part of both the economy and culture, had the highest production in the years 2018 and 2017 [9]. Still, Portugal is the second-largest producer in the world as a percentage of vineyard area in relation to the total area of the country, achieving 194 thousand ha and 2.10%, losing only to Italy, which exhibits 695 thousand ha and 2.31% of the territory [10]. The third country in the ranking, presenting 967 thousand ha (1.91% of the territory), is Spain, followed by France, which has an area of 786 thousand ha (1.22%).

According to the International Organization of Vine and Wine, Portugal appears as the 11th wine producing country and the 9th exporter in the world [10,11]. Generally, 40% of the wine production wastes are burned in the soil, resulting in greenhouse gas emissions (such as CH_4_, N_2_O, CO, and NO_2_) and heat to the atmosphere, leading to environmental damages [12,13]. Promoting studies on this product will possibly increase the added value of waste generated by wineries around the world [14].

The residues of the winemaking industry could be an interesting lignocellulosic biomass to be used in a biorefinery scheme due to their enriched composition in antioxidant compounds (phenolic compounds derived from lignin processing) [15], xylooligosaccharides with potential use as prebiotics [16], and biofuels from enzymatic hydrolysis and the fermentation of cellulose to ethanol [17].

The aim of this review was the identification of lignocellulosic residues derived from the winemaking industry, as well as their composition and the pre-treatments used for the valorisation of the main fractions into value-added compounds. In addition, different strategies for the integral use of this biomass are addressed within a biorefinery scheme.

## 2. Biomass Residues from Grape and Wine Manufacturing

The harvest of vine and the winemaking process (known as vinification) involves seasonal stages in which a large number of residues (including product enriched with a biodegradable content) are generated [7]. Among these wastes, it is important to highlight the biomass derived from pruning the vines, the grape stalks from the destemming stage, and the grape marc or grape pomace obtained from the pressing stage.

**Figure 1:**
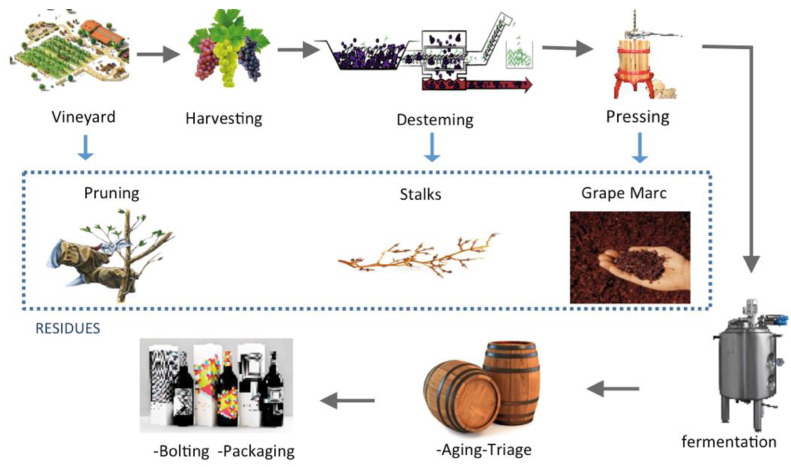
Main stages of vinification, where lignocellulosic residues are generated.

Briefly, grapes are harvested mechanically or manually, stems are removed before fermentation in a process called destemming, and fermentation is carried out by yeasts already present on the grapes (or inoculated). After days of maceration (contact between grape skins and must), the grape must is pressed to separate wine from grape skins, from which grape marc is obtained [18]. These by-products are the main waste generated by the wine making industry, presenting themselves as a raw material of lignocellulosic source of immense potential. Figure 1 summarizes the main stages of vinification, where lignocellulosic residues are generated.

In Portugal, the cultivation of vineyards produces about 222,700 ha year, which generates around a range of 2 to 4 t/ha of cultivation of vine-pruning residue [19,20,21]. From these, around 50% of production is residue and only a small volume is used. About 15% of the grapes produced in Portugal are directed to the wine industry, which generates up to 1.5 million metric tons of solid waste per year and consists of different by-products, namely grape marc, stems, and pruning [12].

Table 1 shows the chemical composition of these lignocellulosic residues derived from the wine industry. As can be observed, the variability in composition is an important aspect of these residues. The seasonality of vine waste availability is also an important feature.

### 2.1. Grape Marc

The grape marc is the main waste of winemaking and accounts for 20 to 30% of the total weight of the grapes [32]. Every 6 L of wine generates approximately 1 kg of grape marc, and it is estimated that, annually, 10.5 to 13.1 million tons are produced [25,33]. Grape marc consists of seeds (38 to 52%), skin (38 to 52%), and residual pulp and stems (2 to 10%) [32,34]. The chemical composition and amount of grape marc depends on several factors such as grape cultivation, pressing process, and fermentation steps [35].

The grape marc contains, on average, 20.8% cellulose, 12.5% hemicellulose, 7.2 to 18.8% proteins, 29.8% lignin, and 4.2 to 7.8% ash (*w*/*w*) (Table 1) [22,23,24]. According to Corbin and co-workers [34], the grape marc composition depends on the grape variety, processing method, environmental conditions, and the proportion of the skin, seeds, and cuttings.

Hijosa-Valsero et al. [36] evaluated the production of mannitol using surplus must and wine lees as a carbon source using three species of Lactobacillus (*Lactobacillus fermentum* CECT 285, *Leuconostoc mesenteroides* CECT 8146, and *Lactobacillus intermedius* NRRL B-3693). The bacteria *Lactobacillus intermedius* NRRL B-3693 reached the production of approximately 69 g/L of mannitol from the red wort and approximately 80 g/L of mannitol from the white wort in 48 h of fermentation, and these results reached yields of 0.888 and 0.895 mol/mol, respectively. The yeast extract was replaced by wine lees in order to reduce fermentation costs, which was possible to reach 59.4 g/L of mannitol for red must and 65.6 g/L for white must in 144 h. The compounds extracted from waste of winemaking are of great importance in several sectors (e.g., food, cosmetics, pharmaceuticals, biofertilizer, and energy [35,37,38,39].

### 2.2. Grape Stalk

Grape stalks (the grape cluster skeleton) are an important by-product of the wine industry and are present in the bagasse, representing about 3 to 7.5% (*w*/*w*) of the processed material, with a production of approximately 2 to 3 million tons per year [28,29,40]. The bagasse consists of 30.3 to 36% cellulose, 21 to 24.5% hemicellulose, 17.4 to 34% lignin, 3.9 to 7% ash, 6.1% protein, and 1.7 to 2.3% of extractives [26,41]. According to Pujol and co-workers [41], grape stems are a promising source of valuable natural products. In this sense, some authors have developed studies to obtain polyphenolic compounds, such as adsorbent for the removal of caffeine from aqueous solutions and activated carbon, among others [40,42].

### 2.3. Vine-Pruning Residue

The vine-pruning residue is an abundant lignocellulosic source consisting of a significant percentage of cellulose, and it corresponds to 93% of the by-products generated in the viticulture (Table 1). This residue is typically burnt in the agricultural field, causing environmental pollution [43]. Due to its chemical composition, availability, and low cost, several works have evaluated its valorisation by different processes to obtain several products such as oligosaccharides from hemicellulose, antioxidant compounds from lignin, organic acids, and bioethanol from saccharification of cellulose, ashes, proteins, and extractives (following the biorefinery approach) [17,20,44].

## 3. Fractionation of Lignocellulosic Biomass for its Revalorisation: A Biorefinery Approach

### 3.1. Pre-Treatment as First Step of a Biorefinery for Valorisation of Vine-Pruning Residues

**Figure 2:**
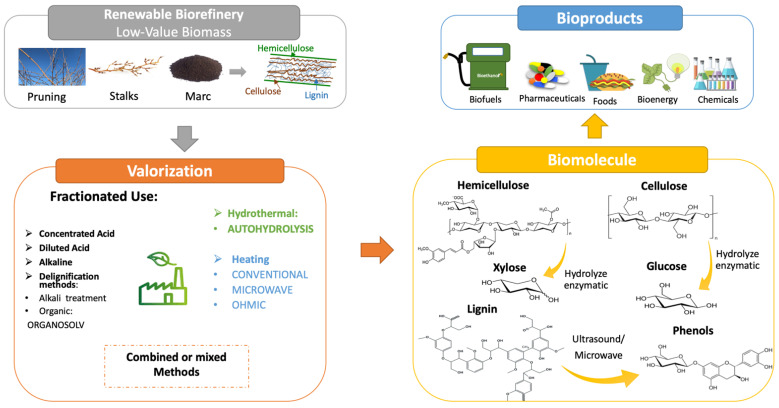
Valorisation of lignocellulosic biomass generated in wine processing following the biorefinery approach.

The bioconversion of lignocellulosic biomass into biofuels, biochemicals, and biomaterials by a biorefinery scheme is one of the potential approaches for sustainable growth based on a suitable use of renewable sources. The integral use of lignocellulosic materials can be divided into two groups: global use (integrated use without previous separation) and fractionated use (selective separation of lignocellulosic components previously to its use). Figure 2 displays the valorisation of lignocellulosic materials following the biorefinery approach.

The global use includes methods of combustion, gasification, pyrolysis, and liquefaction. Combustion is a chemical reaction between two or more reactants (fuels and oxidants) with a release of energy in the form of heat. Gasification is based on the chemical transformation of solid or liquid fuels into a synthesis gas, capable of being ignited immediately for energy production or serving as raw material. Pyrolysis is based on the thermal decomposition of the lignocellulosic material in the controlled presence of gases (H, CO, CO_2_, and hydrocarbons of low molar mass), resulting in liquid (gaseous condensation product) and solid (biochar) fractions. Liquefaction is based on the conversion of a gas into a liquid.

On the other hand, the fractionation approach of a lignocellulosic biomass is encouraged as an alternative to the global use since it allows use of their main fractions (cellulose, hemicellulose, and lignin). Nevertheless, the main challenge is component separation without chemical degradation. These processes can be classified considering the main fraction obtained: delignification methods (solubilization of lignin), hydrolysis of polysaccharides (solubilization of polysaccharides), and a combination of both.

The first step in a biorefinery approach is to alter the recalcitrant structure of the lignocellulosic biomass, also known as pre-treatment. A physical, chemical, physico-chemical, or biological pre-treatment plays a significant role in the fractionation of lignocellulosic materials, to obtain suitable fractions for the production of the desired compounds or value-added products.

To follow the concept of biorefinery, a physical treatment must be carried out in advance. This physical treatment consists of reducing the size by milling, or milling drying the biomass, to improve the processing efficiency. Other physical treatments such as ultrasound application, vaporization, extrusion, autoclaving, and voltage electrical discharges are also widely used to alter the structure of the lignocellulosic materials by increasing the surface area of the biomass [45].

Chemical pre-treatment consists of using different catalysts, such as acids, alkali, and oxidizing agents. The most widely used acid for the fractionation of lignocellulosic materials is H_2_SO_4_ [46]. It converts hemicelluloses into simpler sugars. Alkaline pre-treatment involves the use of bases such as NaOH, and has been extensively studied [47]. These are responsible for breaking down the lignin structure of the biomass, leading to cellulose swelling, and partial altering the crystallinity index of cellulose, resulting in improved accessibility of the enzymes to cellulose and hemicellulose.

The physico-chemical pre-treatment consists of thermal treatments such as hot water, autohydrolysis, and steam explosion. This process solubilizes the components of the lignocellulosic material according to the pH, moisture content, and temperature [48].

Biological treatments are based on the use of biological agents, such as fungi, which act as biocatalysts with the function of selectively degrading lignin while preserving cellulose. The enzymatic hydrolysis also has the function of catalysing fermentable sugars from insoluble cellulose or hemicellulose, releasing it into the medium [17,49,50]. The objectives are to increase the surface area and porosity of the substrate, reducing the crystallinity of cellulose and disrupting the heterogeneous structure of the cellulosic materials. So far, no single method of pre-treatment was found to meet all these requirements; instead, a combination of different methods can be applied [51,52]. An “ideal” pre-treatment should satisfy the following requirements:

1. Simple and economical operation;s
2. Limited requirements of energy, process water, and chemicals;
3. Limited corrosion;
4. Ability to alter the structure of lignocellulosic material;
5. Selectivity towards polysaccharide losses;
6. High recovery of valuable hemicellulose-derived products;
7. Limited production of undesired degradation products;
8. Production of substrates with high cellulose content and susceptibility towards enzymatic hydrolysis;
9. Generation of high-quality lignin or lignin-derived products; and
10. Limited generation of waste.

With this approach, the residues of wine making have been used as raw materials to produce value-added products as xylitol, ethanol, and antioxidant compounds. Vine-pruning residue (VPR) is a promising lignocellulosic biomass due to its high sugar content, low cost, and abundance. Most free phenolic compounds are present in cell vacuoles. However, it is in the cell wall that lignin, flavonoids, and insoluble polyphenols are conjugated to sugars, carbohydrates, organic acids, proteins, and polysaccharides. However, access to these compounds is required to carry out one or more pre-treatments, generally followed by enzymatic hydrolysis to release the fermentable sugars [17]. Table 2 reports published works on the processing of vineyard waste and the resulting products.

### 3.2. Phenolic Extraction and Composition of Vine Pruning

Despite representing a small fraction, the extractives have high commercial value due to their pharmacological potential. Furthermore, they are mainly composed of phenolic and volatile compounds, including stilbenes (*ε*-viniferin, trans-resveratrol, and trans-piceid) that have been extensively explored in VPR, flavonoids (catechin, epicatechin, vanillin, luteolin, hesperidin, quecertin, and apigenin), and phenolic acids (caffeic acid, gallic acid, *p*-coumaric acid, ferulic acid, and ellagic acid), which are both present in higher concentrations in some vine varieties [13,17,52,60,61,64].

According to Sánchez-Gómez and co-workers [77], lignin can release low molecular weight phenolic compounds, alcohols, aldehydes, ketones, or acids. In this sense, several studies have employed different current green technologies to obtain bioactive compounds, such as conventional heating (CHE) [51,58], microwave-assisted extraction methods (MAE) [51,57], high temperature hydrothermal treatment [17,54], ultrasonic-assisted extraction (UAE) [57], alkaline hydrolysis [53], pulsed electric field extraction (PEFE) [78], solid–liquid dynamic extraction (SLDE) [58], pressurized solvent extraction (PSE), supercritical fluid extraction (SFE) [60], superheated liquid extraction (SHLE) [57], subcritical extraction of water (SWE) [58], pressurized liquid extraction (PLE) [60,79], and combined treatments such as SFE and PSE [60], alkaline hydrolysis and enzymatic hydrolysis assisted by pre-treatment with physical by high-voltage electric discharges (HVED) [52], and ohmic heating (OH), which are considered good options [62]. In addition, the use of the vine lignin for the extraction of phenolic compounds (ferulic acid, *p*-coumaric acid, and other phenolic compounds) can also be evaluated using a combination of two pre-treatments (acid and alkaline hydrolysis) [80].

The acidic pre-hydrolysis (3% H_2_SO_4_ for 15 min at 130 °C, liquid solid ratio 7.64:1 g/g) allows the conversion of the major polysaccharides into monosaccharides. The resulting solid from the acid pre-hydrolysis is subjected to an alkaline hydrolysis (4–12% NaOH, for 30–120 min, 50–130 °C) to promote delignification. The phenolic profile analysis carried out shows that the majority of the hydroxycinnamate acids used are ferulic and *p*-coumaric with high concentrations (141.0 and 31.5 mg/L, respectively). The hydroxybenzoic acid that presents the highest concentration is gallic acid (164.4 mg/L) [53].

Some authors have used physical treatments such as PEF, HVED, and UAE to extract polyphenol and proteins. There is a significant increase in phenolic compound extraction in both treatments, although at different energy intensities. The treatment that obtains better results with a lower energy expenditure is HVED, with an energy input of 254 kJ/kg, causing greater damage to the cellular structure of the treated VPR. The highest yield of total phenolic content (TPC) is 34.5 mg of gallic acid equivalent (GAE) per g of VPR, with 89% purity and 4.4 mg/g proteins [56].

The physical treatment by HVED is combined in another work with alkaline (delignification) and biological treatments (enzymatic hydrolysis) in VPR to obtain different products, such as polyphenols, reducing sugars, and soluble lignin. The TPC (3.7 mg GAE/g VPR), reducing sugars (110 mg/g VPR), and soluble lignin (1.5%) present higher yields when high voltage electric shocks are applied before enzymatic hydrolysis, which also improves the subsequent delignification process, reducing the lignin content by 10%. This combination of treatments also positively influences the concentration of individual phenolic compounds such as ferulic acid (186 μg/g VPR), resveratrol (26 μg/g VPR), and *p*-coumaric acid (140 μg/g VPR). These results show the efficiency of the use of HVED combined with enzymatic hydrolysis, which favours the extraction of phenolic compounds in the delignification process [52].

In addition, some studies [58] suggest the application of high-pressure technologies in VPR as a good alternative for the extraction of polyphenolic compounds. Some authors [58,60] have evaluated the potential of two varieties of VPR (“Touriga Nacional” and “Tinta Roriz”) for the extraction of bioactive compounds by comparing three different methods: MAE, SWE, and CHE. The “Tinta Roriz” variety shows better results for flavonoids (18.7 mg epicatechin equivalents (EE)/g VPR) in the treatment by subcritical extraction of water, and TPC (32.1 mg GAE/g VPR) for microwave extraction. These methods produce extracts with higher concentrations of bioactive compounds, where the major phenolic compounds identified are gallic acid (175 mg/100 g), catechin (592 mg/100 g), myricetin (281 mg/100 g), and kaempferol-3-*O*-rutinoside (125 mg/100 g).

Other high-pressure techniques were also applied to the extraction of VPR stilbenes, such as SFE and PLE. These extraction methods were compared with the ethanolic extraction in Soxhlet, which reaches 231 mg/100 g of *trans*-resveratrol, 156 mg/100 g of *trans-ε*-viniferin, and 69 mg/100 g of *r2*-viniferin. The stilbene yield obtained by PFE (85–210 mg/100 g trans-resveratrol, 20–92 mg/100 g *trans-ε*-viniferin, and 1–18 mg/100 g *r2*-viniferine) is lower than the yield obtained by PLE and Soxhlet. These differences are attributed to the molecular dimensions where the solubility is reduced according to the dimensions of the solute molecules. The extracts obtained by PLE yield 240–258 mg/100 g of *trans*-resveratrol, which are higher when ethanol is added at 10 MPA and 80–100 °C, with extraction times and solvent consumptions lower than those used in extraction with Soxhlet. However, *r2*-viniferine 10–70 mg/100 g has an equal or greater yield than that obtained in Soxhlet when using temperatures of 100 °C or less. Nevertheless, a decline is observed when the extraction is performed above 100 °C, probably due to thermal degradation. However, when combined with PSE and PFE treatments, it is possible to obtain a yield of 10–172 mg/100 g *trans*-resveratrol, 7–99 mg/100 g *trans-ε*-viniferin, and 0–40 mg/100 g *r2*-viniferine. These values are lower than those obtained by Soxhlet and PLE. Therefore, although high pressure extraction techniques have been shown to be more economical and efficient for the extraction of phenolic compounds of VPR, further studies are necessary to optimize the combination parameters of the methods [60].

It is described in the literature that the hydrothermal treatments can be an environmentally friendly alternative to obtain extracts with a high content of high value-added phenolic compounds. From the extracts obtained by hydrothermal treatments and purified by the ethyl-acetate stage, the yield obtained is between 0.9 and 3.8 g extract/100 g VPR, with a maximum TFC yield of 0.9 g, and routine equivalents (RE)/100 g of VPR and TPC of 1.6 g of GAE/100 g of VPR at a temperature of 215 °C. In the study by Gullón et al. [54], the compounds identified, derived from sugar and lignin, are probably due to the severity of the treatments. The major compounds identified are vanillin (0–3.5 mg/100 g extract), acetovanillone (0–8 mg/100 g extract), guaracyl acetone (0–3.1 mg/100 g extract), syringaldehyde (3.7–3.5 mg/100 g extract), and acetosyringone (2.7–12.9 mg/100 g extract). However, in another study [16] using autohydrolysis extraction at 180 °C, 2.3 GAE g/L of extract were obtained with a yield of 3.1 GAE g/100 g of VPR. These values are higher than those obtained by Gullón et al. [54] when using the temperature of 215 °C and purification by ethyl acetate. This strategy allows the obtention of a refined aqueous phase enriched with oligosaccharides, which can be applied without being used as a prebiotic, and is an organic phase composed of polyphenolic compounds with high antioxidant activity [81]. The extracts consisted mainly of hydroxycinnamic acids, as well as hydroxybenzoic acids, flavonoids, and stilbenes. However, the compounds with the highest concentrations are catechin (47.5 mg/L extract), chlorogenic acid (34.7 mg/L extract), caffeic acid (33.6 mg/L extract), rutin (20.6 mg/L extract), syringic acid (18.8 mg/L extract), resveratrol (12.4 mg/L extract), rosmarinic acid (12.2 mg/L extract), *p*-coumaric acid (10.3 mg/L extract), gallic acid (8.4 mg/L extract), cinnamic acid (7.2 mg/L extract), and ferulic acid (5.0 mg/L extract). Based on these results, it can be stated that the severity of the treatments may influence the TPC concentration and the phenolic profile of the VPR extracts [17].

In the search to optimize the parameters (ethanol concentration, extraction time, and temperature) for the extraction of bioactive compounds of VPR using environmental friendly methodologies and using non-toxic solvents as hydroalcohol mixtures, a study of the extraction was carried out using the CHE method [51]. For CHE extraction, it is possible to reach the maximum concentrations using 45% ethanol for 120 min at 80 °C, while extracting TPC of 2.17 GAE g/100 g VPR. However, for MAE extraction, it is possible to reach the maximum concentration of TPC (2.3 g/100 g of VPR) using 60% ethanol for 5 min at 120 °C. The major compounds found in CHE and MAE extraction are ellagic acid (68.6 and 185.1 mg/100 g VPR, respectively) and apigenin (208.23 and 118.8 mg/100 g VPR, respectively). These results show that the treatments by MAE can reduce the time of extraction and, consequently, the energy consumption, proving to be an efficient technology for the extraction of polyphenolic compounds. This also allows for selective extraction of specific compounds, accordingly to the conditions applied to the process of extraction. Therefore, extracts obtained by microwave treatments in hydroalcoholic solutions have a high content of ellagic acid and apigenin and interesting antioxidant activity, highlighting the different methods [51].

Recently, ohmic heating was used for the extraction of phenolic compounds in vine prunings. The extracts obtained by OH in two different intensities of electric fields (LEF refers to low electric field and IEF to intermediate electric field) showed 12 different types of polyphenolic compounds, where the ones with the highest concentrations were apigenin (384.2–157.5 mg/100 g VPR), quercetin (287.2–286.8 mg/100 g VPR), ellagic acid (222.9–77.7 mg/100 g VPR), and hesperidin (180.3–149.0 mg/100 g VPR) in IEF and LEF, respectively [64]. Therefore, the extraction of bioactive compounds from VPR are widely studied. However, there are several factors that can influence its chemical composition, such as cultivars, geographic location, climatic conditions, soil characteristics, and storage time [63].

As previously shown, VPRs contain a large number of phenolic compounds which are of great interest for industrial and pharmacological applications. The results reported in the literature show that the extracts obtained from VPR stand out over other lignocellulosic biomasses (olive pruning and eucalyptus leaves, among others) [82,83]. Among the stilbenes groups that are widely reported in VPR and grapevine by-products, trans-resveratrol is the most abundant, with interesting biological activities such as antioxidant, anti-aging, anti-inflammatory, hepatoprotective, and estrogenic/antiestrogenic activities (used for aging in post-menopausal women, also acting in the control of metabolic and cardiac disorders) [84,85,86,87,88,89,90,91]. In addition, phenolic compounds such as quecertin, apigenin, and ellagic acid were also widely detected in VPR, and these compounds have a great biological capacity (antioxidant, anti-cancer, anti-inflammatory, and antimicrobial, among others), making this raw material more valuable. Quercertin is a flavonoid widely studied as an anti-cancer agent, which can prevent different types of cancer. In addition, some studies show that this compound has no effect against normal cells and can be used as an efficient therapeutic agent for various types of cancer [92,93].

Although apigenin has been little described in VPR, there are some studies that have detected it in high concentrations. This flavanol has great antioxidant activities with analgesic potential, as well as anti-cancer, anti-inflammatory, and healing properties [94]. Furthermore, the presence of this compound has been described in grape pomace [95]. Ellagic acid is a phenolic acid abundant in VPR which has pharmacological properties, and there are several studies to evaluate its potential for prebiotic, anti-inflammatory, antioxidative, anti-inflammatory, and anti-apoptotic activity [96,97,98,99]. These compounds make VPR extracts a potential agent in the treatment and prevention of numerous diseases.

### 3.3. Applications of VPR Extracts in Agriculture and Pharmacological Properties

**Figure 3:**
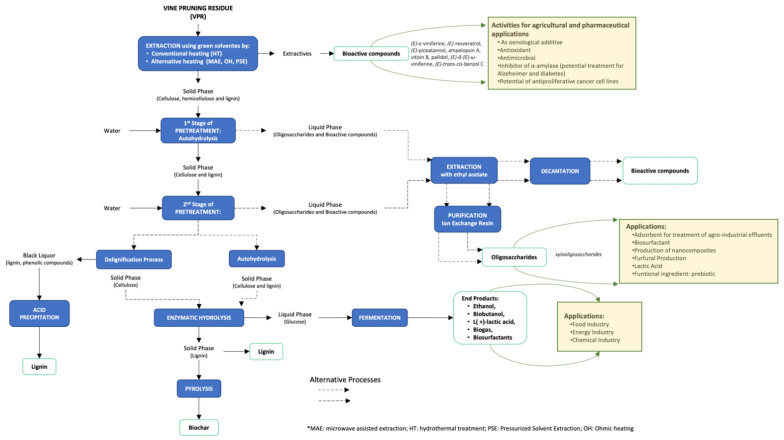
Proposal of a biorefinery scheme for the integral valorisation of vine-pruning residues and the potential applications of the main components.

Due to their great potential, the bioactive compounds of the VPR were explored in search of different and innovative applications (Figure 3). Some authors have proposed the aqueous extracts of VPR (“Airén” variety) by CHE as a wine biostimulant, which are efficient as foliar fertilizers and increase the amino acid content of the wine [64]. Other studies have evaluated the efficiency of the aqueous extracts of VPR (“Muscat” variety), previously treated by toasting as leaf biostimulants in the “Arién” variety, where it is observed that the production of grapes presents higher yield, producing wines with lower alcoholic content and with higher contents of odours activity, for norisoprenoid compounds (*β*-damascenone), vanillin derivatives (vanillin and acetovanillone), and volatile phenols (guaiacol and syringol) [65].

Cebrián-Tarancón and co-workers [63] evaluated the release of the phenolic compounds of VPR (“Airén” and “Cencibel” varieties), roasted and unroasted, in two concentrations (4 and 12 g/L) during hot maceration times in wine models composed of ethanol/water (12.5/87.5 *v*/*v*) and 5 g/L of tartaric acid, adjusted to pH 3.5 with 1 m NaOH, where the concentrations of vanillin or guaiacol were higher than the wine odour limits, depending on the variables applied, as well as high concentrations of trans-resveratrol and ellagic acid. Due to the high transfer rate of compounds, it can be said that the VPR has oenological capacity that can increase the sensorial and functional quality of the wines.

Another study [100] evaluated the effect of the VPR addition of the varieties “Airén” and “Cencibel” as oenological additives. The VPR were previously crushed in pellets and granules in the concentration of 12 g/L and put in contact with wines during different vinification stages. In this study, the (−)-epicatechin and (+)-catechin compounds were elevated in comparison to the control wines. For the white wine “Airén”, guaiacol and *trans*-resveratrol had higher concentrations, and an increased concentration of 4 mg/L. As for the red wine “Cencibel”, the compound that presented the highest concentration was vanillin, which was four times higher than its odour threshold in all grapevine varieties at all times of addition, independent of the grape variety, shape, and moment of addition. β-ionol was detected only in wines in which VPR was added after the fermentation process. In this sense, the use of VPR as an innovative alternative to modulate the chemical composition of wines, elaborating different wines, enables the connection of viticulture and oenology in a new concept of circular viticulture [63].

The antifeedant activity of the aqueous extract of VPR was investigated [101] in the insects *Spodoptera littoralis*, *Leptinotarsa decemlineata*, and *Myzus persicae*. The study proved that the extracts presented significant antifeedant activity using *Lolium perenne* seeds and stimulated the root lengthening of *Lactuca sativa*.

Over the years, the emergence of drug-resistant microorganisms in different environments has been increasing, which has reduced the efficiency of antibiotics. Due to the significant presence of high-value compounds of VPR extracts, some authors [54] have evaluated extracts obtained by hydrothermal treatments and extraction with ethyl acetate for its antioxidant potential and antimicrobial activity against six strains of Gram-positive and -negative bacteria (*Listeria innocua*, *Staphylococcus aureus*, *Escherichia coli*, *Bacillus cereus*, *Pseudomonas aeruginosa*, and *Salmonella* spp.). From extracts obtained by hydrothermal treatments and extraction with ethyl acetate with extract yield between 0.95 and 3.80 g of extract/100 g of VPR, the identified compounds were vanillin, acetovanillone, guaracyl acetone, syringaldehyde, and acetosyringone. The antimicrobial activity of the extracts presented a minimal inhibitory concentration and minimal bactericidal concentration (between 5–20 mg/mL). Thus, the results obtained showed that VPR extracts obtained by autohydrolysis and extraction by ethyl acetate showed great potential of bioactive compounds with antimicrobial and antioxidant activity [54].

Other authors [58] have evaluated the inhibition of α-amylase, antioxidant activity, and antimicrobial activity of the extracts of VPR (varieties “Tinta Ruiz” and “Touriga Nacional”) by MAE, SWE, and CHE against Gram-negative bacteria (*Escherichia coli* and *Escherichia coli* ESA37, resistant to cephalosporins), Gram-positive bacteria (*Streptococcus mitis* and *Streptococcus mitis* ESA65 from lactam resistance), and the yeasts *Candida albicans* ATCC 10231 and *C. albicans* from amphotericin resistance. The “Touriga Nacional” variety was associated with higher antioxidant activities, with FRAP values of 24.3 mg of ascorbic acid equivalent (AAE)/g of VPR and of DPPH of 35.3 mg of Trolox (TE)/g VPR, both for the extracts obtained by SWE. However, for the protective effect on haemolysis induced by AAPH2,2′-azobis-2-amidinopropane and the antimicrobial activity, the extracts of the variety “Touriga Nacional” obtained by MAE showed better results. These ranged from 0.6–4.0 mg of extract/mL for minimal inhibitory concentration, 1.5–6.0 mg extract/mL for minimal lethal concentration, and IC_50_ of 9.59 μg/mL for inhibition of oxidative haemolysis [58].

Thus, the results showed that the VPR extracts present great potential as bioactive compounds with antimicrobial, antioxidant, and inhibitory activity against the α-amylase and acetylcholinesterase enzymes, and they could be used as adjuvants in the treatments of diseases such as Alzheimer’s and diabetes. In addition, the extracts obtained through MAE extraction present better results in comparison with autohydrolysis, although this treatment showed higher concentrations of total phenolic compounds. This difference is probably due to the phenolic composition present in each extract as a result of the severity of the treatment. The extracts obtained by autohydrolysis presented higher concentrations of lignin-derived compounds, whereas in the extracts obtained by MAE extraction, the majority of the compounds were derived from the cellular wall. Another factor that may have had influence is the synergy of the complex mixture of the bioactive compounds.

A recent study [49] tested the antioxidant and antiproliferative activities of stilbenes isolated from the extracts of *(E)-ε*-viniferine, *(E)*-resveratrol, *(E)*-piceatannol, ampelopsin A, vitisin B, pallidol, *(E)-δ-(E)-ω*-viniferine, *(E)-trans-cis*-benzol C, isorhapontigenin, scirpusin A, and a new isomer called isoscirpusin A. The results obtained showed that the oligostilbenoids isolated from VPR have potential benefits to health [61].

Jesus et al. [62] reported that the VPR extracts obtained by OH showed inhibition of the fungi *Alternaria* sp., *Cladosporium cladosporioides*, *Phoma violacea*, *Penicillium italicum*, and *Penicillium expansum*. The results obtained for the antifungal activity varied according to the concentrations and extracts; however, none of the concentrations showed 100% inhibition. The antiproliferative potential was also evaluated in four different cancer cell lines (MDA-MB-231, MCF-7, HepG2, and Caco2) and one non-cancer cell line (CCD 841 CoN) using the MTT method [50]. Similar to what happened with the antimicrobial analysis, all VPR extracts inhibited cell growth according to extract concentration and exposure time for all cell lines tested. However, the IEF extract showed a greater inhibition efficiency against MDA-MB-231, MCF-7, HepG2, Caco2, and CCD 841 CoN cells with IC_50_ values after 48 h of exposure of 62.8, 54.7, 89, 7, 49.7, and 71.0 μg/mL, respectively. These results can be attributed to the great potential of the compounds (quercetin, apigenin, ellagic acid, and hesperidin) present in the extracts, which provided the selective reduction of viability on cancer cells.

Subsequently, Jesus and collaborators [102] evaluated the anti-human colorectal cancer capacity of the IEF extract and the extract together with the chemotherapy drug 5-FU. In that study, it was possible to verify that the extract IEF reduced the proliferation of rectal cancer cells, providing DNA effects and cell cycle modulation. It also increased the sensitivity of cells to the chemotherapy drug 5-FU. The study suggested the use of the extracts as functional food additives or nutraceuticals that could be explored in personalized, anti-colorectal cancer dietary strategies.

### 3.4. Application of Polysaccharides from Vine Pruning in the Production of Bioproducts

There are reports in the literature [71] proposing the use of oligosaccharides obtained by the acid hydrolysis (using mineral acids such as H_2_SO_4_ in an autoclave at 130 °C) of vine pruning in the production of adsorbent compounds for the treatment of agro-industrial effluents. In these reports, it was verified that the non-encapsulated VPR hydrolysate removed 27.8% of the dyes, while the in the case of calcium alginate, the spheres removed 77.3%. In view of these results, it can be stated that VPR are a potential biocomposite in the treatment of industrial effluents.

Other authors [66] have studied the production of glycolipopeptide biosurfactants by *Lactobacillus paracasei* from the hemicellulosic fraction obtained by acid pre-treatment (3% H_2_SO_4_; 15 min at 130 °C, liquid–solid ratio of 8:1 *w*/*w*), followed by alkaline delignification (6.5% NaOH; 60 min at 130 °C; liquid–solid ratio of 10:1 *w*/*w*), where they obtained about 70.7% cellulose, 1.7% hemicellulose, and 25.5% lignin. After the enzymatic hydrolysis, the glucose yield was 47% and there was an ability to reduce the surface tension of 25.1 mN/m and a minimum concentration to maintain the lower surface tension of an aqueous solution of 1.35 mg/mL of biosurfactant. These values represent an advantage from an industrial point of view due to their greater efficiency.

The fraction of VPR cellulose previously fractionated by alkali treatments (4 wt% NaOH solution at 80 °C for 2 h) and the fibers obtained in this process by bleaching three times in acetate buffer (27 g NaOH and 75 mL glacial acetic acid) and aqueous sodium chlorite (1.7%), followed by an acid hydrolysis (NaOH) process, were also experimented upon for the production of cellulose nanocrystals regarding the development of nanocomposite materials. The obtained cellulose nanocrystals presented a needle shape with an average length of 456 nm and an average diameter of 14 nm, and had a high crystallinity index (82% and thermal stability compared with nanocomposites obtained from other raw materials and the same process of extraction, as well as a greater capacity of reinforcement, raising the mechanical resistance of nanocomposites based on biopolymers [49].

Other authors have evaluated the acid and alkaline treatments for the best recovery of cellulose from the VPR, allowing an efficient enzymatic hydrolysis. The pre-treatment conditions used were acidic H_2_SO_4_ 41% (120 °C for 30 min), which allowed the removal of 69% of hemicellulose and obtained a recovery of 96% of cellulose, followed by alkaline treatment with 3% NaOH at 120 °C without stirring for 60 min, which recovered 75% of cellulose and 25% of lignin. However, for the improvement of enzymatic hydrolysis efficiency, it was necessary to carry out a milder pre-treatment of the biomass with 2% NaOH at 100 °C to achieve a glucose yield of 98.72% with 35.06 g L and 31.9% of lignin [68].

Recent studies [21] used a catalysed medium (water/1-butanol/H_2_SO_4_) in a microwave technology as an alternative treatment to solubilize the lignin and hemicellulose of VPR in order to obtain a solid rich in cellulose. In this study, it was possible to solubilize 85% of lignin of good purity, resulting in a solid of 75% cellulose. After the treatment, the lignin was separated from the medium and the hemicellulose derivatives of the aqueous phase were converted into 64.6% furfural.

In another study, Garita-Cambronero and co-workers [46] applied alkaline and acid pretreatments (with and without washing steps) to obtain VPR sugars. The best results were obtained for the recovery of sugars (40.21 g/L) when using acid pre-treatment with 1.7% H_2_SO_4_, 134 °C for 17 min with 10% *w*/*w* VPR, followed by enzymatic hydrolysis. These values are within the range described by other authors for VPR [75]. After saccharification, three thermotolerant strains of *Bacillus coagulans* DSM 2314, *Geobacillus stearothermophilus* DSM 2313, and *G. stearothermophilus* DSM 494 were tested for lactic acid production under aerobic and non-sterile conditions. The strain that showed the best results in fermentation was *B. coagulans* DSM 2314, which was able to produce approximately 29 g/L of lactic acid in 24 h, reaching a sugar consumption of 99 and a yield of 96% when supplemented with the lees of red wine, without the need for detoxification steps.

Recent studies [16] have investigated the probiotic activity of VPR oligosaccharides obtained by autohydrolysis treatment (severity of 4.69), concentrated by ultrafiltration (1 kDa to the concentration ratio by volume of 5.1) and purified by ion exchange resin (IRA-96). The purified oligosaccharides (99%) were submitted to gastrointestinal digestion in vitro, simulating the three stages of digestion (mouth, gastric digestion, and small bowel conditions) and the prebiotic effect (fermented in vitro with human faeces). The results showed that the oligosaccharides obtained are resistant to gastrointestinal digestion. However, when the undigested mixture is subjected to fermentation in human faeces for 48 h, it was verified that 80% of the oligosaccharides were consumed. In addition, the *Bifidobacterium* population increased by 14%. These results demonstrated efficiencies in the use of VPR oligosaccharides treated as a prebiotic constituent with functional properties important for human health. Finally, some of the potential applications of oligosaccharides derived from vine-pruning hemicellulose are shown in Figure 3.

### 3.5. Potential of Vine Pruning for the Production of Biofuels

Previous studies have shown that VPR was also exploited as an energy source for boilers in the wine industry, replacing pine pellets with VPR chips. These have a high heating value (18 MJ/kg on a dry matter basis) [72]. Combustion tests were carried out in a biomass boiler to evaluate the viability of stable steady state combustion and the corresponding environmental impact. The gases emitted during combustion presented values lower than the limits required by the European legislation; however, the particles were higher than the established limit. Nevertheless, the large and medium sized boilers have systems of traces that prevent the release of these particulates to the environment, a technology that does not exist in household equipment. The results showed that VPR pellets can be used for energy conversion in medium and large biomass boilers, and they are not feasible for domestic use. Ashes produced during the coarsening process showed high levels of copper, with the same limits for use as agricultural fertilizer in some European countries. Thus, to make VPRs a viable alternative in economic and energy terms, it is necessary to develop environmentally friendly technologies for the valorisation process.

Methodologies such as pyrolysis lead to the significant production of combustible gases and are a good source of graphite carbon, with higher calorific value. Due to the high lignin content, grape pruning in nature is an excellent raw material for the production of biochar. Some authors [74] have studied the effects of pure CO_2_ and N_2_ use at two absolute pressure levels (0.1 and 1.1 MPa) at 600 °C of pyrolysis of VPR. They found that pyrolysis in CO_2_ and N_2_ atmospheres yields a similar fixed carbon and biochar mass. In a CO_2_ atmosphere, the CO_2_ yield was reduced; however, the CO_2_ yield increased. Therefore, these results indicate that the recycling of CO_2_ obtained by the combustion of VP as the pyrolysis atmosphere is a promising substitute for N_2_, which is a relatively expensive gas.

Other authors [73] have evaluated the production of VPR biochar, obtained by physical and chemical activation, using CO_2_ and KOH, where the CO_2_ absorption capacity at different temperatures (0.25 and 75 °C), apparent CO_2_ selectivity on N_2_, and isosteric adsorption heat were tested. The charcoal activated chemically at 25 °C and presented a higher adsorption capacity of CO_2_, overcoming results obtained in similar works with other lignocellulosic materials. However, the coals prepared with physical activation at 800 °C during 1 h of immersion presented a higher adsorbent capacity of CO_2_ with high adsorption rates at higher temperature. Based on these results, it can be stated that VPR biochar is a promising material that can also be used as an efficient adsorbent with several applications, such as CO_2_ after combustion, biogas upgrading, and H_2_ purification.

For the production of biofuels through lignocellulosic biomass, it is necessary to perform different pre-treatments (such as hydrolysis) to break down the recalcitrant lignin structure, aiding the enzymatic and microbial accessibility [17,30,47,51], as shown in Figure 3. The combination of methodologies such as UAE and enzymatic hydrolysis were suggested as pre-treatments for biogas production through anaerobic digestion. However, methane and biogas yields were higher for VPR treated only with enzymatic hydrolysis [50].

Garita-Cambronero and collaborators [75] studied the feasibility of producing biobutanol by fermentation of acetone-butanol-ethanol in VPR. To obtain butanol, the pre-treatment adopted was alkaline hydrolysis (1.16% NaOH (*w*/*w*), 125 °C, and 110 min) and subsequent enzymatic hydrolysis, a strategy adopted to reduce the concentration of phenolic inhibitors and increase the recovery of fermentable sugars. These conditions allowed obtaining 42.84 g/L of total sugars corresponding to 65.21% recovery and 0.84 g/L of phenolic compounds. The hydrolyzate produced was subjected to fermentation by 11 strains of Clostridium, where the highest concentration of butanol (8.0 g/L) was obtained by the strain *C. beijerinckii* CECT 508.

As for the production of bioethanol, some authors used pre-treatments by alkaline hydrolysis of VPR, followed by enzymatic hydrolysis and fermentation for the production of glucose as a source of carbon for the production of bioethanol [47]. The results indicate that the reaction time and the NaOH concentration influence the response, where the optimum glucose yield (202 g glucose/kg of VPR) was reached at 2.5% NaOH for 40 min at 120 °C. In the search for environmentally friendly methodologies, some studies have been developed using innovative technologies that apply thermal and water treatments, followed by enzymatic hydrolysis, to obtain bioethanol [47]. The authors proposed the use of pre-treatments such as steam explosion [70], autohydrolysis [69], and two sequential hydrothermal treatments [17] to improve the the enzymatic saccharification of VPR for the production of bioethanol.

Some authors [70] have used pre-treatments by VPR vapour blast, followed by enzymatic hydrolysis and fermentation, for the production of glucose as a carbon source. According to Buratti and co-authors, the treatment of the vapour extraction in the severity of 4.56 is efficient for the production of bioethanol, obtaining maximum of 8.9 kg of ethanol/100 kg of VPR, and referring to a yield of 81.09% of ethanol.

Therefore, this work shows that VPR is a promising biomass for the production of biofuels, energy, and chemical products, as it is a renewable and low-cost alternative to petroleum. Previous studies have shown that vine pruning has a higher xylan and glucan content than those found in other residues from the wine industry [29] and from other parallel industries, such as olive tree pruning from the olive oil industry [103,104,105,106]. However, compared with other lignocellulosic materials, namely hardwood (such as eucalyptus and paulownia), the reported results obtained from VPR were quantitatively lower due to the higher glucan content in hardwoods (approximately 44%). Nevertheless, the extraction of phenolic compounds during biomass pre-treatment using vine-pruning residues is higher than using eucalyptus wood [83,107,108]. Regarding the recalcitrant structure of VPR, when compared with other lignocellulosic materials, this could be very similar to the behaviour of hardwoods, taking into account results obtained after pre-treatment on the enzymatic susceptibility of cellulose and the composition of hydrolysates enriched mainly by xylooligosaccharides [107,109,110].

### 3.6. A Biorefinery Approach for the Integral Valorisation of Vine-Pruning Residues

Currently, the biorefinery concept is encouraged for the valorisation of residues obtained from the agricultural and food industries in order to achieve a sustainable development based on a bioeconomy. Considering the above-mentioned chemical composition of VPR, several value-added products can be obtained using different biomass pre-treatments. In this sense, it is necessary to develop biorefinery configurations to promote the efficient separation of their components, as well as their further conversion into chemical compounds and derivatives.

This approach has been recently applied by several authors [17,69] that have developed an integrated process of fractionation using hydrothermal treatments, followed by enzymatic hydrolysis for the production of bioethanol, xylooligosaccharides, lignin, and phenolic compounds from VPR. For instance, Dávila et al. [69] evaluated the sequential processes of hydrothermal treatment (with a severity of 4.47) followed by an alkali delignification process for ethanol production (13.3 g/L) and the recovery of high-quality lignin from black liquor. On the other hand, Jesus and co-workers [17] proposed sequential autohydrolysis stages for the recovery of oligosaccharides in the liquid phase and the improvement of the enzymatic saccharification of the pre-treated biomass, achieving 99% of cellulose to glucose conversion and 13.1 kg of ethanol per 100 kg of VPR in the most suitable conditions.

Thus, through the studies developed so far for the full recovery of VPR, it is possible to consider an integrated biorefinery approach, based on different processes, to obtain a cascade of multi-products (Figure 3). In this context, the process would be initiated by an extraction using a heating method, combined with hydroalcoholic mixtures, to obtain bioactive compounds with a high antioxidant power, as performed in previous works [52,81,85]. In addition, the solids resulting from the extraction could be the subjects of two sequential autohydrolysis treatments, or, alternatively, autohydrolysis, followed by a delignification process to recover xylooligosaccharides, with antioxidant activities in the liquid phase and a susceptible cellulose to enzymatic hydrolysis [17,69]. To separate the xyloligosaccharides from the bioactive compounds, the liquid phase can be subjected to an ethyl acetate extraction process [54].

The solid resulting from the two autohydrolysis processes can be explored in different ways. They can be subjected to acid hydrolysis to obtain nanocrystalline cellulose, or saccharified through enzymatic hydrolysis. After conversion, the glucose can be fermented, thus giving rise to different products such as ethanol, biobutanol, L(+)-lactic acid, biogas, and biosurfactants. At this point, the solids resulting from the lignin-rich fermentation process can be subjected to pyrolysis and transformed into biochar. On the other hand, other authors have suggested the valorisation of the solid fraction resulting from the fermentation process for the production of wood bioethanol and grape stems as a source of raw material for the production of cellulose nanocrystals [28,111].

Thus, the biorefinery concept presented is suggestive for the full recovery of vine-pruning residues, making these promising substrates for the economic production of bioethanol, xyoligosaccharides, bioactive compounds, and lignin, among others. These studies show that it is possible to make the recovery of waste from the wine industry, leading to a reduction in production costs and waste avoidance, in accordance with the principles of the circular economy.

## 4. Conclusions

The composition of lignocellulosic biomass may vary depending on geographic location, climate, process, and variety of vine studied. There are several lignocellulosic biomass conversion studies of derived wine residues, and these are here being reviewed. Still, there are some fractionation studies of wine residues related to the extraction and production processes of phenolic extracts and antioxidants. Nevertheless, a biorefinery approach could be addressed to obtain added-value products from the different fractions of residues generated in the winemaking industry. The main limitation on the use of lignocellulosic materials obtained from the winemaking industry is to know their chemical composition and to use a suitable pre-treatment for the proposed objective.

This review addresses the most recent studies that use the valorisation of VPR as a raw material for the production of products of industrial interest, as well as their perspectives, their potential, and future challenges. The main limitations encountered were the diversity of processes and products found and the lack of standardization of the units of measurement, which increased the complexity of the comparisons. Based on the analyses, it was possible to observe that there is a need to find an effective, clean, and economically viable methodology for the effective fractionation of VPR. VPR is a high value-added by-product, and the development of an effective fractionation methodology promises to make possible the full use of all the existing fractions within an integrated biorefinery. This would allow directing the refined products to specific industrial sectors, and thus contributing to the circular economy.

## Figures and Tables

**Table 1:** Composition of lignocellulosic material (in %) and annual production (in million tons) present in winemaking waste.

Residues	Cellulose	Hemicellulose	Lignin	Ashes	Proteins	Extractives	Estimated Production	Estimated Availability in World (Million t/Year)	Refs.
**Grape marc**	20.8	2.5	29.8	4.2–7.8	12.1–18.8	39.1	20–30%	10.5–13.1	[22,23,24,25]
**Stalks**	20–36	21–24.5	17.4–34	3.9–7	6.1	1.7–2.3	3–7.5%	2.2–3.1	[26,27,28,29]
**Vine prunings**	32.9–39.9	5.8–27	26.7–46.8	2.6–3.3	2.0–2.7	3.1–16.6	-	2–4	[17,19,21,30,31,32]

**Table 2:** Treatments of VPR that lead to valorisation into different products following the concept of biorefinery.

Treatments	Products	Reference
CHE and MAE *	Phenolic compounds	[51]
Acid and alkaline hydrolysis	Phenolic compounds	[53]
Autohydrolysis	Phenolic compounds	[54]
CHE * and β-cyclodextrin	Phenolic compounds	[55]
Alkaline hydrolysis and HVED *	Phenolic compounds	[56]
SHLE, MAE, and UAE *	Phenolic compounds	[57]
CHE and MAE *	Phenolic compounds	[58]
MAE *	Phenolic compounds	[59]
Enzymatic hydrolysis, alkaline hydrolysis, and HVED *	Phenolic compounds and proteins	[52]
PSE and PFE *	Phenolic compounds	[60]
CHE *	Phenolic compounds	[61]
OH *	Phenolic compounds	[62]
-	Oenological additives	[63]
CHE, SLDE, PSE, and MAE *	Viticultural bio stimulant	[64]
CHE *	Foliar fertilizer	[65]
Autohydrolysis	Prebiotic oligosaccharides	[16]
Hydrolysis acid	Biosurfactants	[66]
Dilute acid hydrolysis, delignification, and enzymatic hydrolysis	Biosurfactants	[67]
Combined acid and alkali followed by enzymatic hydrolysis	Glucose	[68]
Organosolv processing followed by microwave irradiation	Furfural	[21]
Two sequential stages of autohydrolysis followed by enzymatic hydrolysis	Bioethanol, xylooligosaccharides, phenolic compounds, and lignin	[17]
Autohydrolysis and delignification followed by enzymatic hydrolysis	Bioethanol and lignin	[69]
Steam explosion and enzymatic hydrolysis	Bioethanol	[70]
Alkaline hydrolysis and enzymatic hydrolysis	Bioethanol	[47]
Alkaline hydrolysis	Biocomposite	[71]
UAE * followed by enzymatic hydrolysis	Biogas	[50]
Alkaline hydrolysis, bleaching, and acid hydrolysis	Cellulose nanocrystals for nanocomposite materials	[49]
-	Wood chips and ashes	[72]
Pyrolysis	Ultra-microporous adsorbents	[73]
Pyrolysis	Biochar	[74]
Alkaline hydrolysis followed by enzymatic hydrolysis	Biobutanol	[75]
MAE *	Oligosaccharides, lignin, and cellulose	[76]
Alkaline and acidic hydrolysis, followed by enzymatic hydrolysis	L(+)-lactic acid	[46]

* conventional heating (CHE), microwave-assisted extraction methods (MAE), ultrasonic-assisted extraction (UAE), pulsed electric field extraction (PEF), solid–liquid dynamic extraction (SLDE), pressurized solvent extraction (PSE), supercritical fluid extraction (SFE), superheated liquid extraction (SHLE), subcritical extraction of water (SWE), pressurized liquid extraction (PLE), ohmic heating (OH), and high-voltage electric discharges (HVED).

## Data Availability

Not applicable.
